# Antimicrobial resistance programs in Canada 1995-2010: a critical evaluation

**DOI:** 10.1186/2047-2994-1-10

**Published:** 2012-02-14

**Authors:** John M Conly

**Affiliations:** 1 Infection Prevention and Control, Foothills Medical Centre, Alberta Health Services, Calgary and Area, 1403-29th Street NW, Calgary, T2N 2T9, Canada; 2 Department of Medicine, Faculty of Medicine, University of Calgary, 3330 Hospital Drive NW, Calgary, T2N 4N1, Canada; 3 Department of Microbiology, Immunology and Infectious Diseases, Faculty of Medicine, University of Calgary, 3330 Hospital Drive NW, Calgary, T2N 4N1, Canada; 4 Department of Pathology & Laboratory Medicine, Faculty of Medicine, University of Calgary, 3330 Hospital Drive NW, Calgary, T2N 4N1, Canada; 5 Calvin, Phoebe and Joan Synder Institute of Infection, Immunity and Inflammation, Faculty of Medicine, University of Calgary, Calgary, T2N 4N1, Canada

**Keywords:** antimicrobial resistance, stewardship, antibiotic, scripts, prescribing, population, program components

## Abstract

**Background:**

In Canada, systematic efforts for controlling antibiotic resistance began in 1997 following a national Consensus Conference. The Canadian strategy produced 27 recommendations, one of which was the formation of the Canadian Committee on Antibiotic Resistance (CCAR). In addition several other organizations began working on a national or provincial basis over the ensuing years on one or more of the 3 identified core areas of the strategy. Critical evaluation of the major programs within Canada which focused on antimicrobial resistance and the identified core components has not been previously conducted.

**Findings:**

Data was collected from multiple sources to determine the components of four major AMR programs that were considered national based on their scope or in the delivery of their mandates. Assessment of program components was adapted from the report from the International Forum on Antibiotic Resistance colloquium. Most of the programs used similar tools but only the Do Bugs Need Drugs Program (DBND) had components directed towards day cares and schools. Surveillance programs for antimicrobial resistant pathogens have limitations and/or significant sources of bias. Overall, there has been a 25.3% decrease in oral antimicrobial prescriptions in Canada since 1995, mainly due to decreases in β lactams, sulphonamides and tetracyclines in temporal association with multiple programs with the most comprehensive and sustained national programs being CCAR and DBND.

**Conclusions:**

Although there has been a substantial decrease in oral antimicrobial prescriptions in Canada since 1995, there remains a lack of leadership and co-ordination of antimicrobial resistance activities.

## Introduction

Antimicrobial resistance (AMR) has dramatically increased since the 1990s, and it is widely acknowledged to be a global public health threat [1-4]. In Canada, systematic efforts for controlling antibiotic resistance began in 1997 following a national Consensus Conference held in Montreal entitled "Controlling Antimicrobial Resistance: An Integrated Action Plan for Canadians" [5]. The conference, co-sponsored by Health Canada and the Canadian Infectious Disease Society, developed a plan which emphasized 3 core areas: antimicrobial stewardship, surveillance to monitor resistance trends and infection prevention and control (IPC). The Canadian strategy produced 27 recommendations, one of which was the formation of the Canadian Committee on Antibiotic Resistance (CCAR), a multidisciplinary committee which performed a collating and coordinating role for stakeholder groups across Canada. In addition several other organizations began working on a national or provincial basis over the ensuing years on one or more of the 3 identified core areas formulated during the Consensus Conference. Critical evaluation of the major programs within Canada focused on antimicrobial resistance and the identified core components has not been previously conducted. This paper describes the identification of major AMR programs in Canada between 1995 and 2010 and critically examined the components of surveillance and stewardship.

## Methods

Data was collected from multiple sources to determine the components of four major AMR programs that were considered national based on their scope or in the delivery of their mandates, including the Canadian Committee on Antibiotic Resistance (CCAR), Do Bugs Need Drugs (DBND) - originating in the province of Alberta and adopted in the provinces of British Columbia and Saskatchewan, National Information Program on Antibiotics (NIPA) and the National Collaborating Centre for Infectious Diseases (NCCID), whose mission is to protect the health of Canadians by facilitating the use of evidence and emerging research on infectious diseases to inform public health programs and policy. Sources of data collection for Antimicrobial Resistance (AMR) Programs included the following: review of CCAR Updates in the Can J Med Micro Infect Dis 1998-2009; review of all program websites; and a review of the CCAR led "Pan Canadian Stakeholder Consultations on Antimicrobial Resistance 2009". Assessment of program components was adapted from the report from the International Forum on Antibiotic Resistance (IFAR) colloquium [6]. Through an agreement with Intercontinental Medical Statistics (IMS) HEALTH Canada and its Compuscript database, complete antimicrobial consumption data on all classes of oral antimicrobials in Canada was provided to CCAR up until 2004 and to the Canadian Integrated Program for Antimicrobial Resistance from 2000-2010 [7]. The IMS HEALTH Canada Compuscript database provided continuous surveillance data of the total number of antibiotic prescriptions dispensed in Canadian retail pharmacies based on a representative sample of 2000 pharmacies stratified by province, store type and size [7]. Population data by year was collected from Statistics Canada [8]. Data sources for surveillance of key marker organisms was collected from multiple sources including a publication by the CCAR International Report Card Working Group [9] and a survey of multiple Canadian websites that reported the results of surveillance data. Critical evaluation and assessment of bias of surveillance with respect to reporting, objectives, host population, sampling, population demographics, organisms, isolate collection, susceptibility testing, data handling and analysis was retrieved from Stephen et al. [9]

## Results

A summary of the components of four major AMR programs that were profiled are provided in Table 1. Although the DBND Program was initially provincial in scope, it was included in the evaluation since other provinces began using the program or portions of the program. Three of the programs had governmental funding and one was funded by a pharmaceutical company and had a very short duration. Most of the programs used similar tools but only the DBND Program had components directed towards day cares and schools. Only the CCAR Program distributed "toolkits" to all Canadian physicians. The DBND program had the most rigorous evaluation of its activities.

**Table 1:** National AMR Programs in the Community in Canada 1995-2010

	CCAR	DBND	NIPA	NCCID
Years	1998-2009	From 1998	2001-2006	From 2008
Scope	National	Provincial	National	National
Duration	12 yrs	Ongoing	5 yrs	Ongoing
Funding	Federal Gov't	Provincial	Pfizer	Federal Gov't
Public Communications				
Pamphlets/brochures	Yes	Yes	Yes	Yes
Press conferences	Yes	Yes	Yes	Yes
Posters	Yes	Yes	Yes	Yes
Television/Radio	No	Yes	Yes	No
Video (eg, clinic room)	No	Yes	No	No
Website	Yes	Yes	Yes	Yes
Day-care programme	No	Yes	No	No
School programme	No	Yes	No	No
Professional Communications				
Doctors/Pharmacists/Nurses	Yes*	Yes	Yes	Yes
Scientific journal articles	Yes	No	Yes	Yes
Treatment guidelines	Endorsed DBND	Yes	No	No
Letters to doctors	Yes	Yes	No	No
Toolkits Distributed	Yes^†^	No	No	No
Educational outreach	Yes	Yes	No	No
Feedback	No	Yes	No	Yes
Undergraduate curriculum	Yes	Yes	No	No
Antibiotic prescription pads	Yes	Yes	Yes	No
Symptomatic therapy scripts Evaluation	Yes	Yes	Yes	Yes
Controls for evaluation	No	Yes	No	No
Patient/physician knowledge	No	Yes	Yes	No
Antibiotic use	Yes‡	Yes	No	No
Resistance rates	Yes	Yes	Yes	No
Clinical outcomes	No	No	No	No

CCAR: Canadian Committee on Antibiotic Resistance; DBND: Do Bugs Need Drugs?; NIPA: National Information Program on Antibiotics; NCCID: National Collaborating Centre for Infectious Diseases

*Included information for veterinarians

^† ^all 56,000 physicians in Canada

^‡ ^obtained agreement with IMS HEALTH Canada to provide up-to-date national and regional antibiotic consumption data through its Compuscript database

The sources of antimicrobial resistance surveillance, their funding source and sources of bias are illustrated in Table 2. Unfortunately all the surveillance programs have limitations and/or significant sources of bias. Although the CIPARS surveillance is the most comprehensive, it is not population based and focuses on pathogens associated with food-borne illnesses [10].

**Table 2:** Antimicrobial Resistance Surveillance in Canada

*Program name*	*Funding*	*Focus*	*Assessment*
Canadian Integrated Program for Antimicrobial Surveillance (CIPARS) http://www.phac-aspc.gc.ca/cipars-picra	PHAC	Food borne pathogens Antimicrobial usage	Comprehensive Reliance on passive reporting for Salmonella
Canadian Nosocomial Infection Surveillance Program (CNISP) http://www.phac-aspc.gc.ca/nois-sinp/survprog_e.html	PHAC	Nosocomial pathogens (MRSA, VRE, ESBL)	Focused on tertiary care
Canadian National Centre for Streptococcus http://www2.provlab.ab.ca/ncs/ncs.htm	PHAC	Group A streptococci	Not representative
Canadian Tuberculosis Laboratory Surveillance System http://www.phac-aspc.gc.ca/publicat/tbdrc01/index.html	PHAC	*M. tuberculosis*	
Canadian Bacterial Surveillance Network (CBSN) http://microbiology.mtsinai.on.ca/research/cbsn/default.asp	Pharma	*S. pneumoniae* *H. influenzae*	Biased sampling Potential COI
Canadian Antibiotic Resistance Alliance (CARA) http://www.can-r.com	Pharma	*S. pneumoniae* *H. influenzae *Miscellaneous	Biased sampling Potential COI

PHAC: Public Health Agency of Canada; COI: Conflict of interest

**Figure 1:**
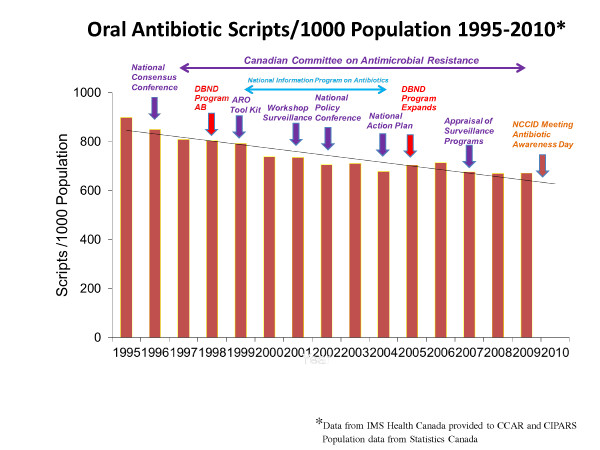
**Oral antimicrobial scripts in Canada and temporal relationship to AMR Programs**.

**Figure 2:**
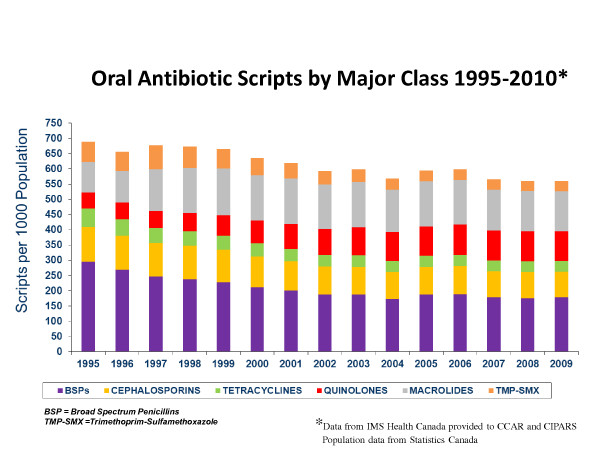
**Oral antimicrobial prescribing by major class in Canada 1995-2010**.

The surveillance results of total oral antimicrobial scripts in Canada, adjusted by population and their temporal relationship to AMR Programs between 1995-2010 and the breakdown of scripts by class are illustrated in Figures 1 and 2. Most of the programs used similar tools but only the DBND Program had components directed towards day cares and schools. Most of the programs used similar tools but only the DBND Program had components directed towards day cares and schools. There appears to be a corresponding increase in the use of quinolones and macrolides, much of this driven by increases in newer generation agents in each of the two categories [10].

## Conclusions

Although the findings from this report focus on only a few nationally focused efforts that took place over the last 15 years, a number of additional provincial, regional and local efforts were also undertaken across Canada [11-14]. Most of the efforts focused on communications to physicians, pharmacists and the general public. Overall, there has been a 25.3% decrease in oral antimicrobial prescribing in Canada since 1995, mainly due to decreases in β lactams, sulphonamides and tetracyclines, in temporal association with multiple programs with the most comprehensive and sustained national programs being CCAR and DBND. It is acknowledged that this is only an association and may be a spurious finding but the consistent and sustained observations over 15 years provide arguments that the AMR programs have had an impact [4].

The findings also demonstrate that there is no population based surveillance of common community pathogens such as *Streptococcus pneumoniae *or *Staphlyococcus aureus *where resistance is common. Reliance on Pharma funded surveillance is not comprehensive and has significant difficulties with sampling bias [9]. There is a need for a federally funded population based surveillance of common community pathogens rather than reliance on Pharma funded surveillance.

Although the CIPARS Program offers a comprehensive and co-ordinated approach to some AMR activities, it is a limited focus. Currently, from the human perspective, there is a distinct lack of leadership and co-ordination of AMR activities at the national level in Canada and concerns may be raised about losing some of the gains that have been made over the years.

## Competing interests

JC has received honoraria from the Canadian Agency for Drugs and Technologies in Health for work as an expert reviewer and clinical expert, respectively, for projects on the role of rapid polymerase chain reaction (PCR) testing for methicillin-resistant *Staphylococcus aureus *in hospitalized patients and the use of vancomycin or metronidazole for treatment of *Clostridium difficile *colitis. He has also received speaker's honoraria related to new antibacterial agents from Janssen-Ortho, Pfizer, and Astellas Pharma during the past five years.

## Authors' contributions

JC was involved in the conception and design of this study, collation of data, the interpretation of the data; drafting the manuscript and revising it critically for important intellectual content; and provided final approval of the version to be published.
